# Comparison of paired cerebrospinal fluid and serum cell‐free mitochondrial and nuclear DNA with copy number and fragment length

**DOI:** 10.1002/jcla.23238

**Published:** 2020-02-13

**Authors:** Aolong Chen, Jun Li, Lei Wang, Qin Huang, Jiajin Zhu, Shumeng Wen, Jianxin Lyu, Wenhe Wu

**Affiliations:** ^1^ Key Laboratory of Laboratory Medicine Ministry of Education Zhejiang Provincial Key Laboratory of Medical Genetics School of Laboratory Medicine and Life Sciences Wenzhou Medical University Wenzhou China; ^2^ Department of Clinical Laboratory Wenzhou People's Hospital Wenzhou China; ^3^ Hangzhou Medical College Hangzhou China

**Keywords:** cell‐free mitochondrial DNA, cell‐free nuclear DNA, cerebrospinal fluid, copy number, fragment length, serum

## Abstract

**Background:**

Most studies on cell‐free DNA (cfDNA) were only for single body fluids; however, the differences in cfDNA distribution between two body fluids are rarely reported. Hence, in this work, we compared the differences in cfDNA distribution between cerebrospinal fluid (CSF) and serum of patients with brain‐related diseases.

**Methods:**

The fragment length of cfDNA was determined by using Agilent 2100 Bioanalyzer. The copy numbers of cell‐free mitochondrial DNA (cf‐mtDNA) and cell‐free nuclear DNA (cf‐nDNA) were determined by using real‐time quantitative PCR (qPCR) and droplet digital PCR (ddPCR) with three pairs of mitochondrial *ND1* and nuclear *GAPDH* primers, respectively.

**Results:**

There were short (~60 bp), medium (~167 bp), and long (>250 bp) cfDNA fragment length distributions totally obtained from CSF and serum using Agilent 2100 Bioanalyzer. The results of both qPCR and ddPCR confirmed the existence of these three cfDNA fragment ranges in CSF and serum. According to qPCR, the copy numbers of long cf‐mtDNA, medium, and long cf‐nDNA in CSF were significantly higher than in paired serum. In CSF, only long cf‐mtDNA's copy numbers were higher than long cf‐nDNA. But in serum, the copy numbers of medium and long cf‐mtDNA were higher than the corresponding cf‐nDNA.

**Conclusion:**

The cf‐nDNA and cf‐mtDNA with different fragment lengths differentially distributed in the CSF and serum of patients with brain disorders, which might serve as a biomarker of human brain diseases.

## 1 INTRODUCTION

The cell‐free DNA (cfDNA), either cell‐free mitochondrial DNA (cf‐mtDNA) or cell‐free nuclear DNA (cf‐nDNA), is released into the body fluid circulation by cells that are in physiopathological conditions, such as necrosis,^1^ apoptosis,^2^ tumors,^3^ inflammation,^4^ pregnancy,^5^ and intense physical exercise,^6^ and it is also present in every healthy people's body fluid. The study on cfDNA has attracted widespread attention because it could be used as a non‐invasive test marker for early diagnosis, diagnosis, and prognosis.^7^, ^8^, ^9^ Many studies have confirmed that cfDNA is present in cerebrospinal fluid (CSF), blood, urine, effusion fluids, and other body fluids.^10^ At present, most of the researches about cfDNA mainly focus on methylation,^11^ copy number variation, fragment length distribution, and mutation rate^10^ in one kind of body fluids. There is little research on the association of differences in copy number and fragment length of cfDNA between different types of body fluids.

Various kinds of techniques have been employed to detect copy number and fragment length of cfDNA. In this study, we utilized Agilent 2100 Bioanalyzer, a device basing capillary electrophoresis, to determine cfDNA fragment length and concentration but requires high demands on the purity and quantity of the samples; otherwise, the protein and ionic contaminants in samples will impact the migration of cfDNA in the capillary.^12^ Then, we utilized real‐time quantitative PCR (qPCR), which is an essential mean for cfDNA quantity detection and commonly applied in scientific research, clinical diagnosis, and progression detection of diseases to determine the copy number of cfDNA.^13^ However, the detection of this method can be impacted by standards preparation and protein contamination in cfDNA samples.^14^ Finally, we used droplet digital PCR (ddPCR), a method that can quantify the absolute number of the target gene given limiting dilutions, PCR, and Poisson distribution for quantifying cfDNA.^13^ Theoretically, ddPCR performs PCR amplification by forming oil droplets, and the amount of contaminant is small containing in each oil droplet. Comparing with qPCR, the ddPCR does not rely on a standard curve and is more sensitive to low‐copy samples detection. However, the qPCR technique is widely used in the determination of cfDNA due to its rapid, inexpensive and straightforward characteristics, and commercial qPCR assays are already available in the quantification of cfDNA.^15^ Moreover, there are still many other pre‐analytical factors affecting cfDNA analysis especially cfDNA extraction in these three methods.^14^, ^16^

Therefore, this study compared fragment length and copy number of cf‐mtDNA and cf‐nDNA by using Agilent 2100, qPCR, and ddPCR in paired CSF and serum of patients who had brain‐related diseases. The concentrations of protein and sodium, potassium, and calcium ions were determined first for evaluating their impacts on the detections for raw and diluted samples. The length distributions of cfDNA fragments were determined by Agilent 2100 for providing references of length on primers designing in qPCR and ddPCR assays. The copy numbers of cf‐mtDNA or cf‐nDNA in different fragment length ranges could be analyzed by the application of multiple primers with different product lengths spanning a target region of mitochondrial *ND1* and nuclear *GAPDH* gene. The sequences amplified by those three primer pairs for either gene are one‐to‐one relationship, that is, the sequence amplified by a shorter‐product primer pair is included in the sequence amplified by a longer one, so a primer pair would amplify any cfDNA template longer than its amplicon, and the shorter one would show a higher readout of copy number than the longer one.^17^ Furthermore, the correlation between qPCR and ddPCR was also analyzed.

## 2 MATERIALS AND METHODS

### 2.1 Subjects and sample preconditioning

The seven patients who were diagnosed as brain‐related disorders such as cerebral hemorrhage, brain trauma, intracranial hypertension, or fever were randomly selected from Wenzhou People's Hospital, and their CSF and serum samples were collected simultaneously. Before splitting and storing at −80°C, the preconditioning steps for CSF and serum samples were performed first. Briefly, the samples were centrifuged at 4°C, 1600 *g* for 10 minutes; then, the supernatants were transferred into new centrifuge tubes and centrifuged at 4°C, 16 000 *g* for 10 minutes to remove cellular debris.^14^, ^18^ The final supernatants that were the raw CSF or serum samples mentioned in full text were dispensed to several tubes and stored at −80°C until use. The freeze‐thaw operation took place in only once to protect cfDNA from degradation.^16^ The study was approved by the Ethics Committee of the hospital, and methodologies conformed to the standards set by the Declaration of Helsinki.

### 2.2 Concentration of protein, Na^+^, K^+^, Ca^2+^

Quantification for the content of protein and metal ions was performed by Enhanced BCA Protein Assay Kit (Beyotime Biotechnology) and PFP7 flame spectrophotometer (Jenway). All operations were executed according to the manufacturer's instructions.

### 2.3 cfDNA fragment length determined by Agilent 2100 Bioanalyzer

To confirm the accuracy of the results and evaluate the potential of utilizing raw CSF and serum samples directly as the sources in Agilent 2100 Bioanalyzer (Agilent Technologies), and to give references of length of cfDNA fragment for qPCR and ddPCR assays, the raw CSF and serum with their serial diluted and extracted samples were prepared. The cfDNA extraction was performed by using TIANamp Micro DNA Kit (Tiangen Biotech)^19^ from 500 μL of raw CSF or serum samples and finally eluted with 20 μL of ddH_2_O. According to the manufacturer's protocol, 1 μL of samples all above mentioned and 1 µL of the ladder were added into disposable chip wells according to the schematic diagram of manual. The markers that contained lower marker 35 bp, 0.125 ng/µL and upper marker 10 380 bp, 0.075 ng/µL, existed in every well, which made the comparison for cfDNA length between different chips feasible.^12^ All of the procedure steps took place in the laminar flow chamber. The analysis of the results did by 2100 Expert software. Analytical specifications of this kit we used ranging from 50 bp to 7000 bp. The length accuracy was ±10% and the size reproducibility was 5% coefficient of variation (CV).

### 2.4 Construction for recombinant plasmids of *ND1* and *GAPDH* gene

The whole genomic DNA template was extracted from peripheral blood mononuclear cells (PBMC), which were obtained from the anticoagulated whole blood of human by Ficol‐Hypaque methods.^20^ The *ND1* gene (538 bp, region: 3441‐3978, *Homo sapiens* mitochondrion, *ND1*, NCBI Reference Sequence: NC_012920.1) and *GAPDH* gene (542 bp, region: 7899‐8440, *Homo sapiens GAPDH*, NCBI Reference Sequence: NG_007073.2) were amplified by PCR. The *ND1* gene represented mitochondrial DNA (mtDNA) and *GAPDH* gene represented nuclear DNA (nDNA). The PCR primers amplifying the *ND1* gene were *ND1*‐F (5′‐ACTACAACCCTTCGCTGACG‐3′) and *ND1*‐R (5′‐GAAGAATAGGGCGAAGGGGC‐3′), while the PCR primers amplifying *GAPDH* gene were *GAPDH*‐F (5′‐TGGTATGAGAGCTGGGGAATG‐3′) and *GAPDH*‐R (5′‐TGGGTGTCGCTGTTGAAGTC‐3′).^14^ These two primer pairs were designed without mutation sites basing on the NCBI gene database. The amplified products of the two genes were separately ligated with pMD18‐T vector through pMD™ 18‐T Vector Cloning Kit (Takara Biomedical Technology) and transformed into chemically competent cell DH5α (Vazyme). Recombinant plasmids were validated by sequencing (Sunny Biotechnology). The plasmid DNA was extracted through Endo‐Free Mini Kit (Omega Biotech), and the concentration was determined by Nanodrop One (Thermo scientific). The molecular weights of *ND1* and *GAPDH* recombinant plasmid DNA were 1 997 071.42 Da and 1 999 592.02 Da separately. The conversion formulas between copy number (*CN*, copies/µL) and concentration (*C*, ng/µL) were as follows:

$$ND1:\;CN^{\text{DNA}}\;\left(\text{copies}/\mu L\right)=\frac{C^{\text{DNA}}\;\left(\text{ng}/\mu L\right)\times 6.02\times 10^{14}}{1997071.42}$$

$$GAPDH:\;CN^{\text{DNA}}\;\left(\text{copies}/\mu L\right)=\frac{C^{\text{DNA}}\;\left(\text{ng}/\mu L\right)\times 6.02\times 10^{14}}{1999592.02}$$

### 2.5 The copy number of cfDNA evaluated by qPCR

Three primer pairs for each recombinant plasmid DNA were designed and applied to evaluate the cfDNA copy number with different fragment lengths by qPCR. As shown in Table 1, these primer pairs are for the external, middle, and inner fragment of *ND1* or *GAPDH* gene sequences binding on plasmids. The sequences amplified by the three primers are one‐to‐one relationships, that is, the sequence amplified by the shorter‐product primer pair is included in the sequence amplified by the longer‐product primer pair. The copy numbers of cf‐mtDNA and cf‐nDNA in CSF and serum were assessed by utilizing SYBR real‐time PCR Master Mix (Vazyme). Briefly, the qPCR was performed with six primer pairs in Table 1 to evaluate above raw samples directly or extracted samples by amplifying the *ND1* gene standing for mtDNA and *GAPDH* gene representing nDNA and to calculate copy number of mtDNA or nDNA from the linearity constructed by dosage dependently standard plasmid DNA solutions. Reaction procedure of qPCR was as follows: 95°C for 3 minutes; 40 cycles at 94°C for 10 seconds, 56°C for 30 seconds, and 72°C for 1 minute.

**Table 1:** Primers of *ND1* and *GAPDH* gene utilized in qPCR and ddPCR

	Name	Sequence (5′‐3′)
External *ND1*	*ND1*‐240bp F	CCCTAAAACCCGCCACATCT
	*ND1*‐240bp R	TTGTTTGGGCTACTGCTCGC
Middle *ND1*	*ND1*‐167bp F	AAAACCCGCCACATCTACCA
	*ND1*‐167bp R	GGATTGAGTAAACGGCTAGGCT
Inner *ND1*	*ND1*‐57bp F	AAAACCCGCCACATCTACCAT
	*ND1*‐57bp R	GTGAGAGCTAAGGTCGGGG
External *GAPDH*	*GAPDH*‐241bp F	CTGAGGCTCCCACCTTTCTCA
	*GAPDH*‐241bp R	CATCACGCCACAGTTTCCCG
Middle *GAPDH*	*GAPDH*‐168bp F	CACCTTTCTCATCCAAGACTGG
	*GAPDH*‐168bp R	CTGTGGTCTGCAAAAGGAGT
Inner *GAPDH*	*GAPDH*‐61bp F	TGGGGACTGGCTTTCCCATAA
	*GAPDH*‐61bp R	CACATCACCCCTCTACCTCC

Abbreviations: F, forward; R, reverse.

### 2.6 The copy number of cfDNA evaluated by ddPCR

The primers and extracted samples applied in ddPCR were as same as in qPCR. Above *GAPDH* recombinant plasmids and 61 bp primer pairs of *GAPDH* gene were utilized for testing the feasibility of the reaction system and procedure of ddPCR assay. Briefly, each of the 20 μL of reactions contained 1× QX200™ EvaGreen ddPCR™ Supermix (Bio‐Rad), 1 µmol/L gene‐specific primers and 2 µL of the template. The ddPCR reaction conditions were as follows: 95°C for 10 minutes; 40 cycles at 94°C for 30 seconds, 55°C for 1 minute; and 98°C for 10 minutes. Droplet readings were executed on QX200™ Droplet Reader (Bio‐Rad), and analysis was performed by Bio‐Rad QuantaSoft software version 1.7.4.

### 2.7 Statistical analysis

Statistical analysis was executed by SPSS 19.0. All the results of copy numbers were converted to log_10_ (copies/µL) in the raw condition before dilution or extraction and data revealed as mean ± SD. The paired *t* test was utilized for copy number difference analysis between paired CSF and serum of all patients. *P* ˂ .05 was considered statistically significant.

## 3 RESULTS

### 3.1 Concentration of protein and metal ions in the patient's CSF and serum

As shown in Table 2, the concentration of protein in serum is much higher than in CSF (*P* < .001), which may be a reason for infeasibility detection by using Agilent 2100 Bioanalyzer and qPCR for raw serum samples. The concentration of Na^+^, K^+^, and Ca^2+^ of raw samples is basically consistent with the clinical detected values (data not shown), and our detected results are listed in Table 3. Our results indicated that there were no significant differences in these metal ions between CSF and serum (*P* > .05).

**Table 2:** Concentration of protein in CSF and serum (mg/mL) (Data are presented as mean ± SD)

Sample type	Patients						
	1	2	3	4	5	6	7
CSF	0.41 ± 0.09	0.66 ± 0.14	0.68 ± 0.1	0.79 ± 0.05	0.94 ± 0.17	1.16 ± 0.1	3.04 ± 0.27
Serum	62.15 ± 3.67	43.44 ± 3.08	51.12 ± 3.16	59.49 ± 1.66	50.29 ± 3.16	51.63 ± 5.81	52.7 ± 3.94

**Table 3:** Concentration of Na^+^, K^+^, Ca^2+^ in CSF and serum (mmol/L) (Data are presented as mean ± SD)

Patients	Na^+^		K^+^		Ca^2+^	
	CSF	Serum	CSF	Serum	CSF	Serum
1	139.94 ± 1.74	122.57 ± 0.63	3.47 ± 0.03	4.21 ± 0.06	2.91 ± 0.07	3.02 ± 0.08
2	142.83 ± 1.44	133.97 ± 2.26	3.17 ± 0.06	4.4 ± 0.06	2.97 ± 0.08	3.1 ± 0.08
3	113.35 ± 3.85	137.59 ± 1.37	2.58 ± 0.08	8.43 ± 0.18	3.23 ± 0.02	3.5 ± 0.08
4	138.67 ± 4.42	134.33 ± 1.37	3.34 ± 0.08	4.54 ± 0.05	3.27 ± 0.03	3.16 ± 0.08
5	131.8 ± 2.19	132.34 ± 2.19	2.49 ± 0.1	4.38 ± 0.09	2.81 ± 0.04	2.79 ± 0.08
6	127.64 ± 2.37	139.57 ± 3.02	2.67 ± 0.02	4.67 ± 0.05	3.25 ± 0.09	3.05 ± 0.1
7	141.75 ± 2.49	136.68 ± 0.31	2.49 ± 0.03	4.66 ± 0.11	3.85 ± 0.09	3.63 ± 0.1

### 3.2 The cfDNA fragment length determined by Agilent 2100 Bioanalyzer

**Figure 1:**
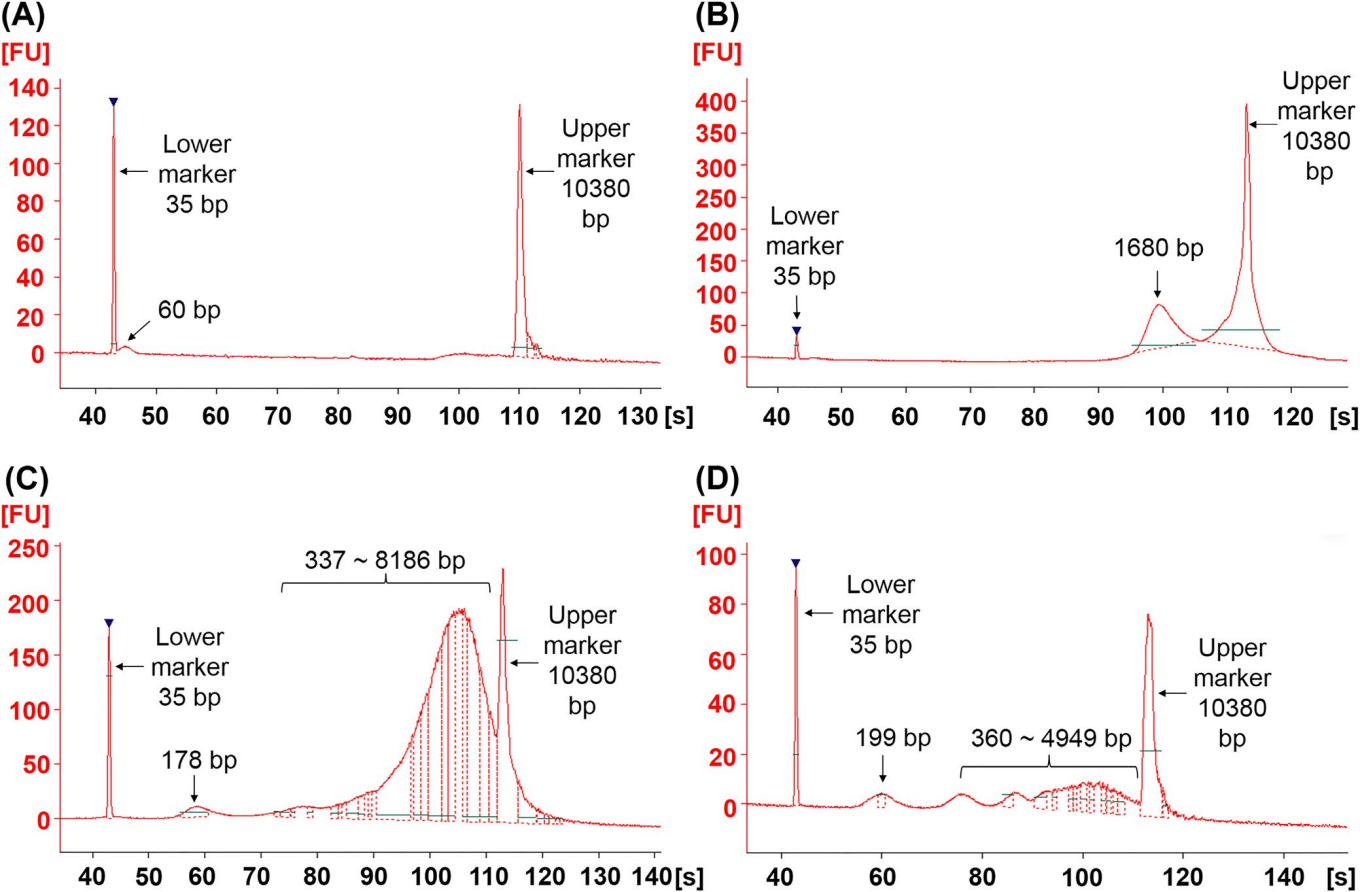
Evaluation of cfDNA fragment length using Agilent 2100 Bioanalyzer. A‐D, Characterization results of raw CSF with fivefold dilution (A), raw serum with fivefold dilution (B), extracted CSF (C) and extracted serum (D) from patient 3. *X*‐axis represents the migration time of DNA fragments. *Y*‐axis indicates fluorescence intensity. The lower marker is 35 bp, and the upper marker is 10 380 bp. The numbers above peaks indicate the length of DNA fragments

To evaluate the fragment lengths of the cfDNA in CSF and serum and design qPCR and ddPCR primers of different fragment length ranges, above seven paired CSF and serum samples without further preconditioning, with dilution, and with DNA extraction were respectively analyzed and compared by using Agilent 2100 Bioanalyzer. Probably because of the existence of protein, the direct detection of raw CSF samples showed a messy baseline but still existed a clear peak in 60 bp and an unsharp peak range of 1495‐7022 bp [a representative example is shown in Figure S1A]. According to published reports,^21^, ^22^, ^23^, ^24^, ^25^ we highly suspected that a range of short cfDNA fragments formed the peak in 60 bp. To reduce the influence caused by protein, serial dilutions of raw CSFs made in ddH_2_O were used as samples for detection, and the baseline became smooth and the unsharp peak became lower along with the dilution factor augmenting, and the short peak in 60 bp could be observed in CSF samples with fivefold dilution (see Figure 1A). However, the concentration of cfDNA was too low to detect in the 10‐fold diluted raw CSF samples (Figure S1C), and we could only find peaks of >1157 bp but no peaks of 60 bp in the raw serum samples without dilution and with 10‐fold or fivefold dilution (Figure S1B,D, and Figure 1B). The cfDNA fragments longer than 250 bp in our results formed probably because the cellular DNA had been released before centrifuging pretreatment due to blood coagulation^16^, ^26^, ^27^, ^28^, ^29^ or the range of long cfDNA fragments had been released from the cells in necrosis.^1^ We assumed that the excessive content of protein directly hindered migrating and displaying of all ranges of short cfDNA fragments except the ranges of long cfDNA fragments near the upper marker. The excessive protein would affect the display of the upper marker, so the cfDNA peaks in the long fragment range near the upper marker might also be affected by protein. We could still see that the peak altitude of the lower marker became higher with the increase in the serum dilution factor, which clarified that the protein existing in serum indeed impacted the migration of short cfDNA fragments. So, we hypothesized that the short and medium ranges of cfDNA fragments of <250 bp might also exist in serum as same as CSF, although we could not observe the peaks of those ranges in raw serum detections.

In order to eliminate the influence of protein and metal ions, the cfDNA was extracted through TIANamp Micro DNA Kit from 500 μL of raw CSF or serum solution and finally eluted with 20 μL of ddH_2_O. As shown in Figure 1C from extracted CSF, medium cfDNA fragments formed the peak in 178 bp, and long cfDNA fragments formed the peaks from 337 to 8186 bp, and these two similar range fragments also could be obtained from the extracted serum (Figure 1D). It is known that 167 bp is considered to be the length of DNA fragments existing together with protein in the form of the nucleosome.^25^, ^30^ The two peaks in 178 bp and 199 bp showing in extracted CSF and serum, respectively, were close to 167 bp, and we suspected that they were formed by the DNA fragments of nucleosome form. However, the short fragment peak of 60 bp could not be seen in both extracted CSF or serum, which might because these short cfDNA fragments embedded less fluorescent dye and lost more easily than long cfDNA fragments during extraction procedure and they were insufficient to form a peak, but the medium and long length cfDNA fragments were enriched during extraction procedure. In conclusion, there were short (~60 bp), medium (~167 bp), and long (>250 bp) cfDNA types totally obtained in the raw CSF and serum and its serial dilution and extraction using Agilent 2100 Bioanalyzer. Hence, it is necessary to design primers to validate the distribution of cfDNA fragments in CSF and serum by using qPCR and ddPCR. Combining with all results for seven paired CSF and serum samples without further preconditioning, with serial dilution, or with DNA extraction obtained by using Agilent 2100 Bioanalyzer and the results concluded from published reports,^21^, ^22^, ^23^, ^24^, ^25^, ^30^ three primer pairs for each recombinant plasmid DNA were designed and applied to evaluate the cfDNA copy numbers of different fragment lengths by using qPCR and ddPCR (for obtaining primer pairs with the best amplification characteristics, the length of the final selected amplicons were slightly different from the three length ranges suggested by Agilent 2100 and published reports). As shown in Table 1, these pairs were for the long (external), medium (middle), and short (inner) fragments of the *ND1* or *GAPDH* gene. These three pairs of primers of the same gene are in an inclusion relationship, that is, the primer pair with shorter amplification length would have a higher detection value of copy number than or equal to the primer pair with longer amplification length in theoretical. By subtracting between the detection values obtained by utilizing different primer pairs, copy numbers of cfDNA for the specific ranges were obtained. So, we defined cfDNA fragment length ranges of 57‐167 bp, 167‐240 bp and >240 bp of the *ND1* gene as the short cf‐mtDNA (S‐cf‐mtDNA), medium cf‐mtDNA (M‐cf‐mtDNA), and long cf‐mtDNA (L‐cf‐mtDNA), respectively. Meanwhile, we defined 61‐168 bp, 168‐241 bp and >241 bp of the *GAPDH* gene as the short cf‐nDNA (S‐cf‐nDNA), medium cf‐nDNA (M‐cf‐nDNA), and long cf‐nDNA (L‐cf‐nDNA), respectively.

### 3.3 The cfDNA copy number evaluated by qPCR

**Figure 2:**
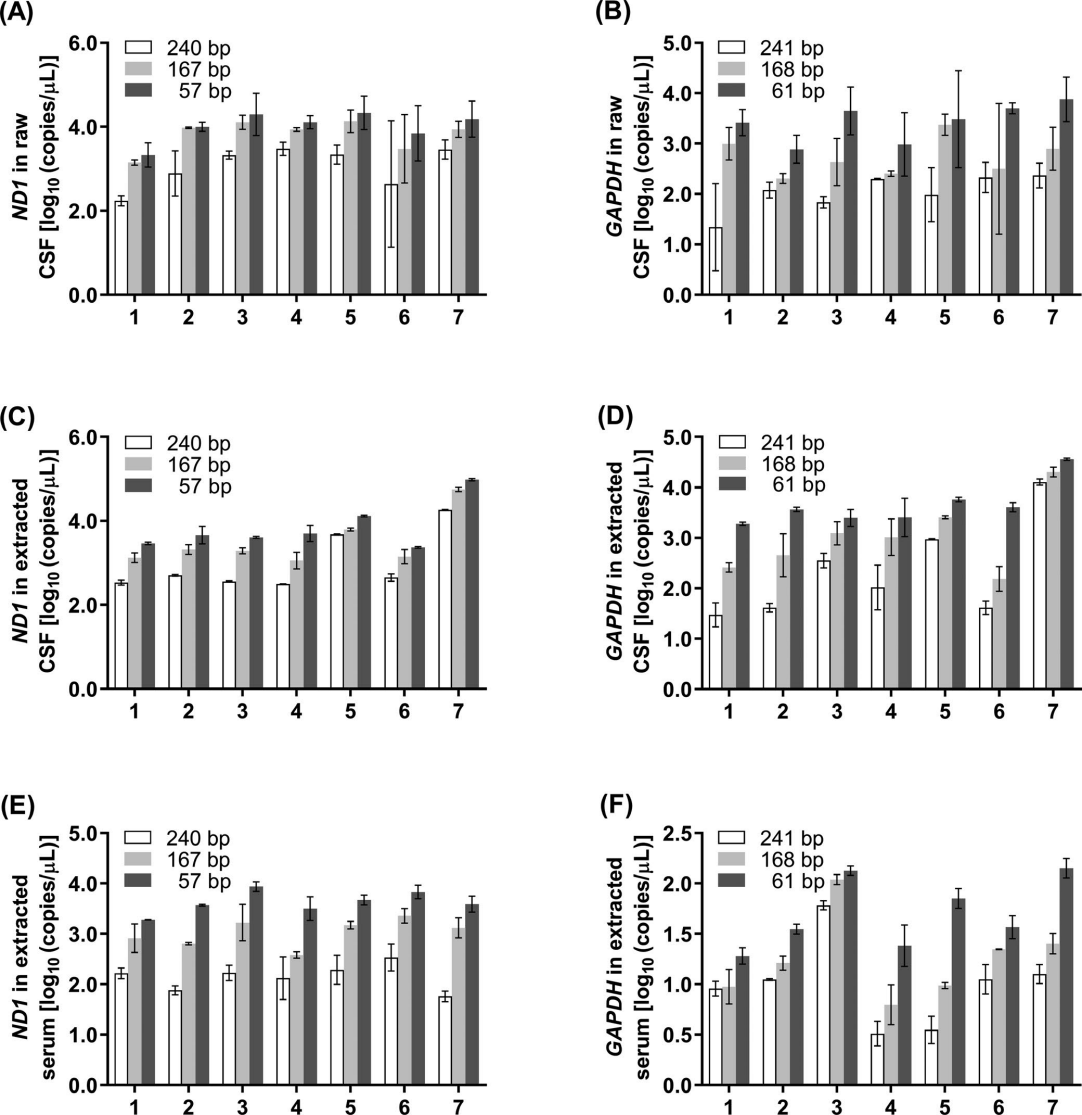
Quantification of cfDNA copy number by qPCR. A, B, Raw CSF was amplified with primer pairs of *ND1* (A) and *GAPDH* (B). C, D, Extracted CSF was amplified with primer pairs of *ND1* (C) and *GAPDH* (D). E, F, Extracted serum was amplified with primer pairs of *ND1* (E) and *GAPDH* (F). *X*‐axis represents patients, and *Y*‐axis represents log_10_ of copies/µL in initial raw samples. Data are presented as mean ± SD, *n* = 3

Recombinant plasmids of the *ND1* and *GAPDH* gene were serially diluted as standards, and the standard curves were established by specific targets and primer pairs (data not shown). These data conform to the publication of qPCR guidelines.^31^ We checked the products of qPCR by electrophoresis to show our primers are correct (Figure S2). To evaluate whether the assay of qPCR was feasible for raw samples detection, we applied all patients' CSF and serum without or with cfDNA extraction as templates for qPCR, and the detection results of extracted samples were converted into the copy number value in raw condition before extraction (copies/µL) and compared with results of raw samples, and all copy number values were through log transformation for plotting. When raw CSFs were used directly as templates (Figure 2A,B), the SDs for replicate groups were more prominent than extracted samples (Figure 2C,D). As shown in Figure S3, there were no significant differences between the data of raw and extracted CSF using all six primer pairs. However, when raw sera were used directly as templates in qPCR assays, the most SDs of Cq value for replicate groups exceeded 0.3 (data not shown), which probably because the excessive protein in raw serum impacted the detection. The cfDNA extracted from 500 µL of raw CSF or serum was performed with six pairs of primers at the same procedure of qPCR. The results revealed that the amplicon with shorter length showed a higher copy number than the longer one in all raw CSF (Figure 2A,B), extracted CSF (Figure 2C,D), and extracted serum (Figure 2E,F), which means, there were some cfDNA fragments whose sequence length were between the shorter and longer amplicons. It indicated that there was cf‐mtDNA or cf‐nDNA of three types fragment length ranges indeed presenting in CSF and serum, which like the results obtained by Agilent 2100 Bioanalyzer.

**Figure 3:**
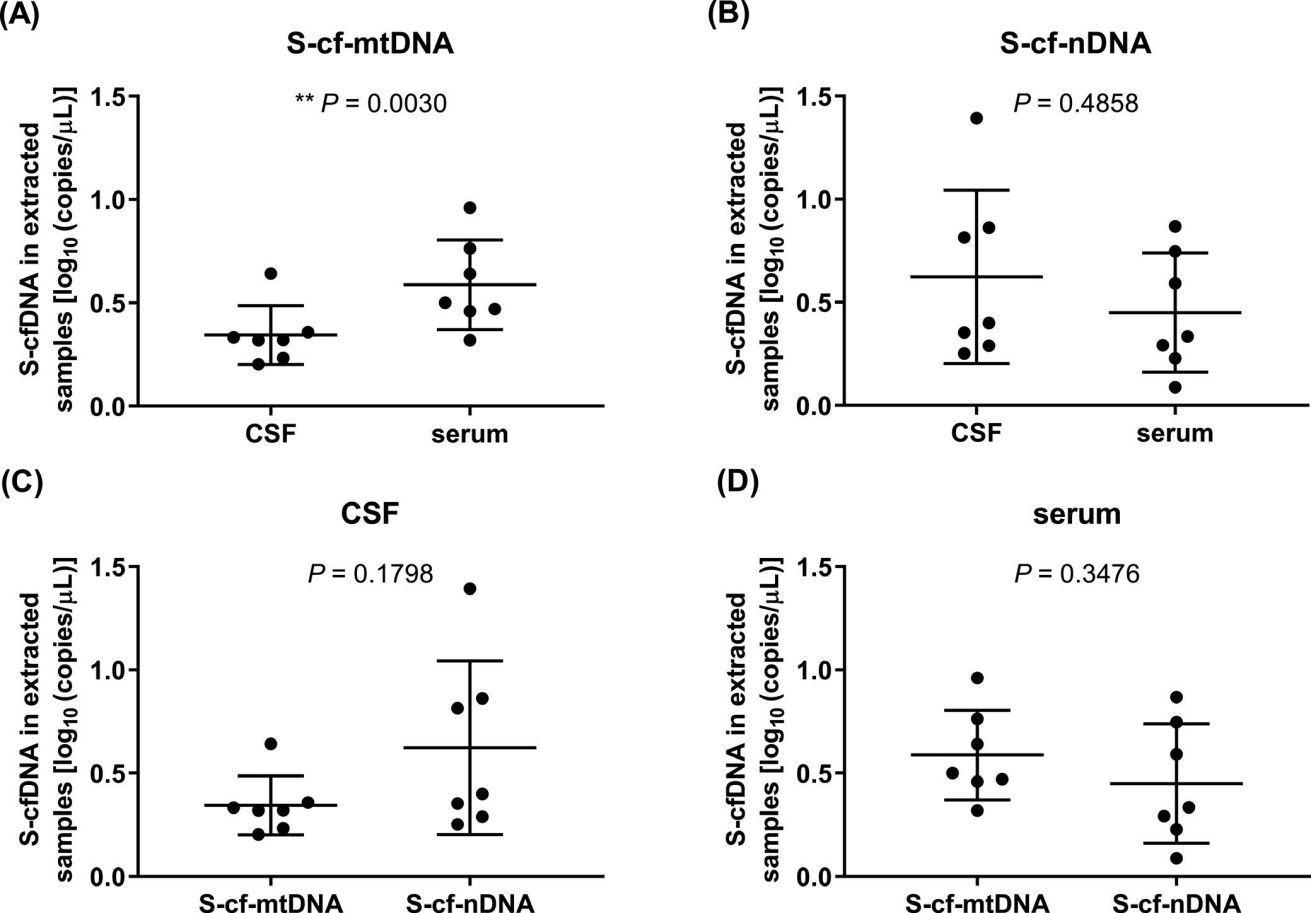
Comparison of copy number of cfDNA in short fragment length by qPCR. A, B, The copy numbers of S‐cf‐mtDNA (A) and S‐cf‐nDNA (B) in CSF and serum. C, D, Copy numbers of S‐cf‐mtDNA and S‐cf‐nDNA in CSF (C) and serum (D). **P ˂ .01. Data are presented as mean ± SD, *n* = 7

**Figure 4:**
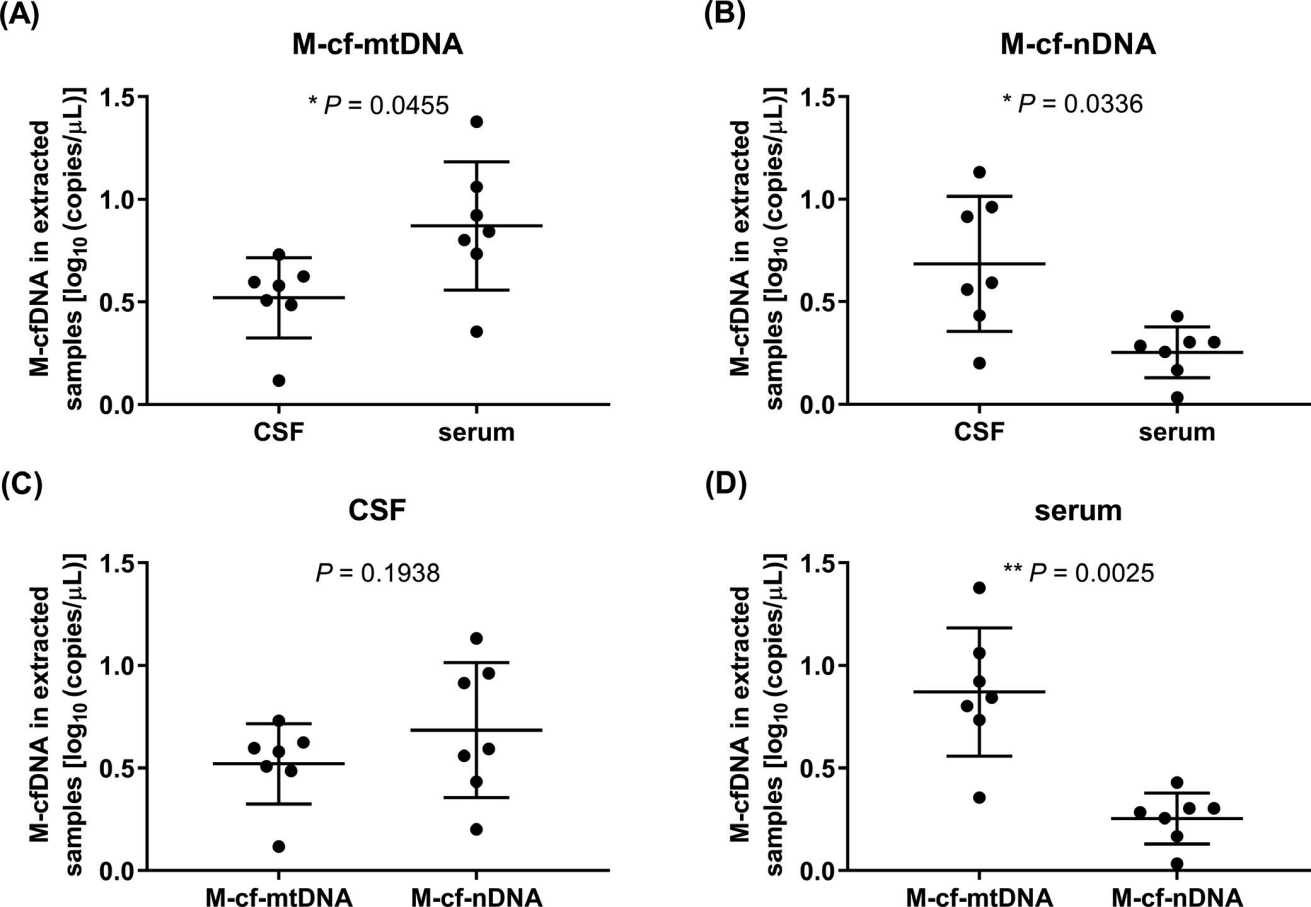
Comparison of copy number of cfDNA in medium fragment length by qPCR. A, B, The copy numbers of M‐cf‐mtDNA (A) and M‐cf‐nDNA (B) in CSF and serum. C, D, Copy numbers of M‐cf‐mtDNA and M‐cf‐nDNA in CSF (C) and serum (D). *P ˂ .05, **P ˂ .01. Data are presented as mean ± SD, *n* = 7

**Figure 5:**
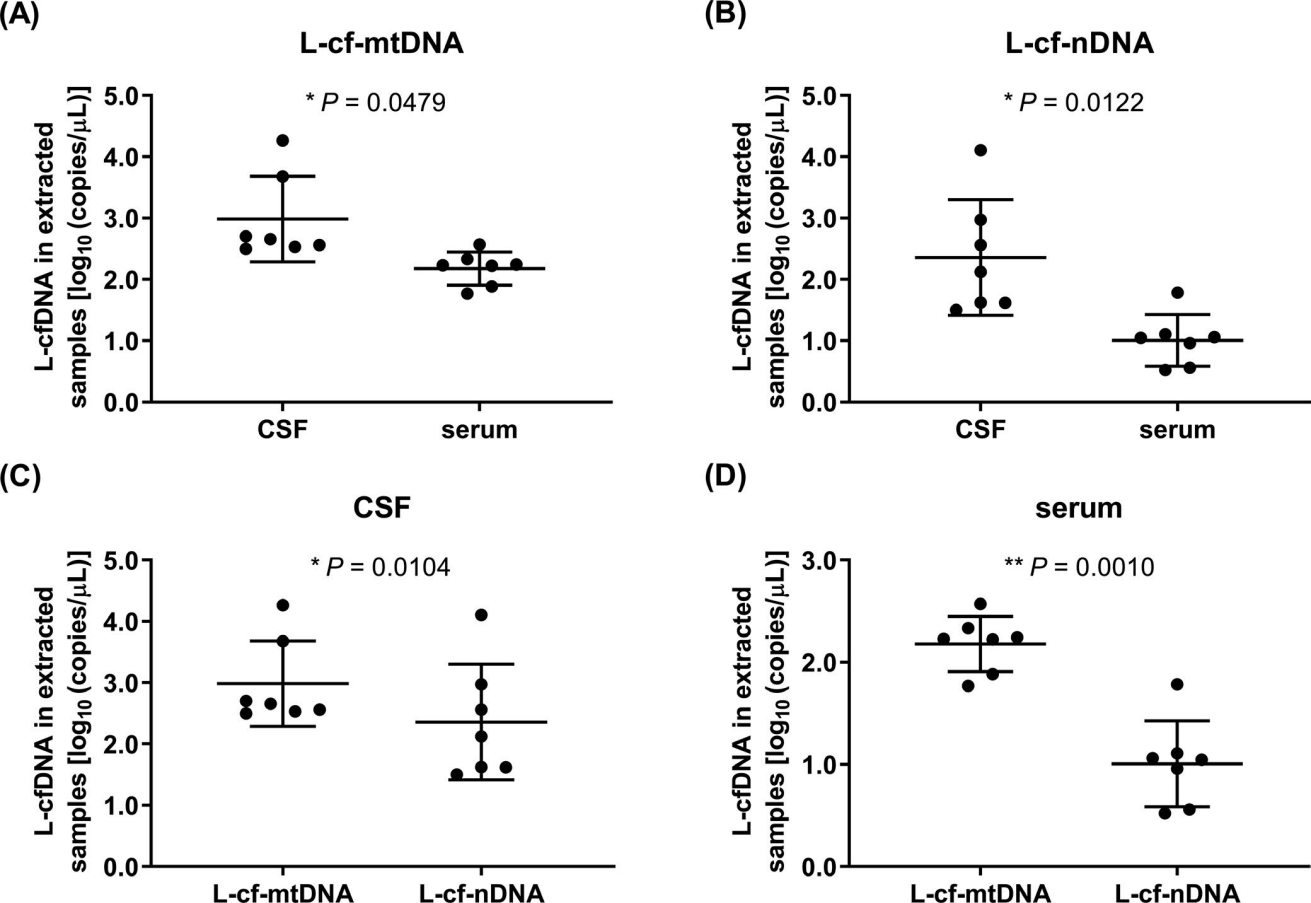
Comparison of copy number of cfDNA in long fragment length by qPCR. A, B, The copy numbers of L‐cf‐mtDNA (A) and L‐cf‐nDNA (B) in CSF and serum. C, D, Copy numbers of L‐cf‐mtDNA and L‐cf‐nDNA in CSF (C) and serum (D). *P ˂ .05, **P ˂ .01. Data are presented as mean ± SD, *n* = 7

By subtracting the copy numbers between different primer pairs, copy numbers with the specific ranges of cfDNAs were obtained (shown in Figures 3, 4, 5). Comparing copy number of cfDNA in short fragment length by qPCR, the S‐cf‐mtDNA was higher in serum than in CSF (*P* = .0030, Figure 3A) but S‐cf‐nDNA was of no significant difference between CSF and serum (*P* = .4858, Figure 3B), and either in CSF (*P* = .1798, Figure 3C) or in serum (*P* = .3476, Figure 3D), there was no significant difference between S‐cf‐mtDNA and S‐cf‐nDNA. As same as S‐cf‐mtDNA, the M‐cf‐mtDNA was higher in serum than in CSF (*P* = .0455, Figure 4A) while M‐cf‐nDNA was lower (*P* = .0336, Figure 4B). Meanwhile, the M‐cf‐mtDNA was also of no significant difference with M‐cf‐nDNA in CSF (*P* = .1938, Figure 4C), but M‐cf‐mtDNA was significantly higher than M‐cf‐nDNA in serum (*P* = .0025, Figure 4D). However, different from the above, both L‐cf‐mtDNA (*P* = .0479, Figure 5A) and L‐cf‐nDNA (*P* = .0122, Figure 5B) were higher in CSF than in serum. And more specific, L‐cf‐mtDNA was higher than L‐cf‐nDNA both in CSF (*P* = .0104, Figure 5C) and serum (*P* = .0010, Figure 5D).

### 3.4 The cfDNA copy number evaluated by ddPCR

Above *GAPDH*‐recombinant plasmids and 61 bp primer pairs of *GAPDH* gene were utilized for the feasibility analysis of the ddPCR assay. The results indicated that this assay had favorable amplification characteristics, an apparent separation between positive and negative droplets (Figure S4A,B), and a good linear correlation in serially standard samples (Figure S4C), which all approved the reliability of this assay using for sample detection. The amounts of total droplets in each well generated during the detection for samples were all above 13 000, indicating a good quality of droplets generation.

**Figure 6:**
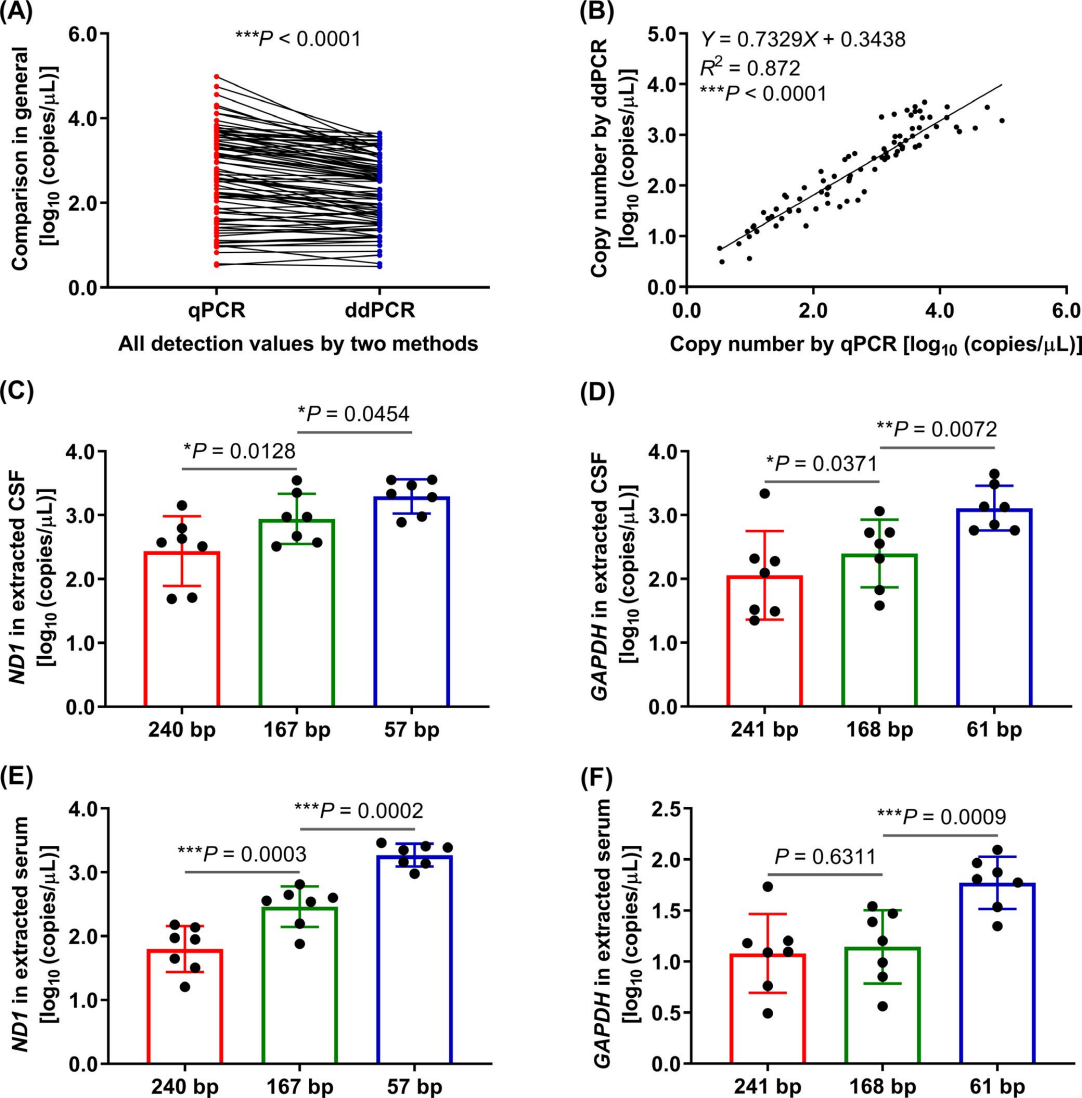
Quantification of cfDNA copy number by ddPCR and comparison with qPCR. A, B, The overall trend (A) and liner correlation (B) between qPCR and ddPCR. All detection values of each method represent that all seven patients' extracted CSF and serum were amplified by all six primer pairs (n = 84), ****P ˂ .0001. C, D, Extracted CSF was amplified with primer pairs of *ND1* (C) and *GAPDH* (D). E, F, Extracted serum was amplified with primer pairs of *ND1* (E) and *GAPDH* (F). *X*‐axis represents primer pairs, and *Y*‐axis represents log_10_ of copies/µL in initial raw samples. *P ˂ .05, **P ˂ .01, ***P ˂ .001. Data are presented as mean ± SD, *n* = 7

In order to analyze the relationship between the qPCR and ddPCR results, we applied all patients' CSF and serum with cfDNA extraction as templates for qPCR and ddPCR detection using all six primer pairs for amplification. Overall, the readout of ddPCR was lower than qPCR (*P* ˂ .0001) (Figure 6A), but there could be a certain consistency (*R* ^2^ = .872) between qPCR and ddPCR according to the correlation equation and the overall trend of the detected values (Figure 6B). Furthermore, from the ddPCR, we could also reveal that the amplicon with shorter length showed a higher copy number than the longer one in most sample detections (93.9%, Figure S5), and most of the higher trends were of significant difference (Figure 6C‐F). These indicated that the short, medium, and long cfDNA fragment ranges indeed existed in CSF and serum and mutually confirmed with Agilent 2100 and qPCR.

## 4 DISCUSSION

Many studies have shown that the plasma is superior to serum on researches of cfDNA, due to the serum itself has a more significant impact on the detection of cfDNA than plasma.^32^ More specific, when the whole blood coagulated in the tube, it caused lysis of white blood cells and releasement of cellular DNA, and this released cellular DNA would affect the determination of cfDNA releasing by cells under physiological or pathological conditions,^16^, ^26^, ^27^, ^28^, ^29^ so the concentration of cfDNA in serum is higher than plasma in theory.^9^, ^33^, ^34^, ^35^, ^36^ Meanwhile, the concentration of different anticoagulants in the plasma will affect the efficiency of PCR.^16^

The patient's CSF and serum samples were collected simultaneously for this study. Our study utilized Agilent 2100 to determine the fragment length of cfDNA and used the qPCR and ddPCR to evaluate the copy numbers of cf‐mtDNA and cf‐nDNA with primers of different amplification lengths. The raw, serially diluted, and extracted samples were all used for detection for getting more information of cfDNA in paired CSF and serum. In order to evaluate whether those contaminants had impacts on the detection of cfDNA, the contents of protein and sodium, potassium, and calcium ions in raw CSF and serum samples were determined. We found that the protein might cause baseline sharp and impacted the readout of small cfDNA fragments in Agilent 2100 results, and it also made the qPCR detection more fluctuant for raw CSF or even undetectability for raw serum. The ion concentration seemed to have little effects on the detection by these three methods.

According to the results of Agilent 2100, we observed three ranges of cfDNA fragments existing in CSF and serum by detecting the raw, serially diluted, and extracted samples. Among these, the short fragment ~60 bp observed in raw and diluted CSF was proved by other studies.^21^, ^22^, ^23^, ^24^, ^25^ But the short peak could not be detected in the raw serum and extracted CSF probably because of the high concentration of protein in raw serum, and less fluorescent dye embedded in these short cfDNA fragments or more easily lost than long cfDNA fragments during extraction procedure in extracted CSF. Refer to published studies,^25^, ^30^ the length of 167 bp is considered to be the nucleosome‐form cfDNA, and we suspected that the medium‐length cfDNA fragments showing in extracted samples were nucleosome‐form cfDNA because they were close to 167 bp. Besides, there were many other cfDNA fragments longer than above two ranges observed in raw, serially diluted, and extracted samples, and among these, the shortest was 337 bp. We guessed these long length ranges of cfDNA fragments formed because the cellular DNA had been released due to blood coagulation before centrifugation pretreatment, and this ranges longer than 250 bp are as published reports.^16^, ^26^, ^27^, ^28^, ^29^

Hence, we designed six pairs of primers basing on the results of Agilent 2100 and amplified the extracted CSF and serum by qPCR and ddPCR to verify the existence of these three ranges of cfDNA fragments. The amplicon lengths of these primer pairs were slightly different from what we concluded through the results of Agilent 2100 and some other published studies before because we made some adjustments in product length to ensure a better primer amplification property, and these cfDNA fragments were basically consistent with other studies.^24^, ^37^, ^38^, ^39^ Many studies proved that the kit produced by QIAGEN company utilized for the extraction and purification of cfDNA and the extraction and purification results were superior to other commercial kits.^23^, ^24^, ^37^, ^40^, ^41^, ^42^, ^43^ Also, more than 1 mL of samples were used for extraction in these studies, which could make the results more accurate and reliable. In summary, there were no significant differences between the data of raw and extracted CSF using all six primer pairs by qPCR. As Agilent 2100 Bioanalyzer, the qPCR and ddPCR results both confirmed that there were short, medium, and long fragment cfDNAs existing in CSF and serum. The detection values obtained from qPCR were higher than ddPCR in general, but the overall trend of qPCR and ddPCR was of a certain consistency. The results of different instruments were roughly but not precisely the same, probably because different instruments had different precisions. Comparing with qPCR and ddPCR, Agilent 2100 does not have a process of DNA amplification. What more, although both qPCR and ddPCR have PCR amplification, the detection principles are not the same.

By subtracting the detection values between different primers in qPCR, the copy numbers of the specific ranges of cfDNAs were obtained. Comparing CSF with serum, the copy numbers of S‐cf‐nDNAs (Figure 3B) as defined above were no significant differences, but either M‐cf‐nDNA (Figure 4B) or L‐cf‐nDNA (Figure 5B) was higher in CSF than in serum; the copy numbers of S‐cf‐mtDNA (Figure 3A) and M‐cf‐mtDNA (Figure 4A) were both lower in CSF than in serum, but L‐cf‐mtDNA (Figure 5A) was higher in CSF than in serum. We know that the cfDNA in body fluids is released by cells, so the cfDNA exists in body fluids under physiological conditions. However, by comparison, it was found that the differences in copy number of cfDNAs between CSF and serum were not the same among different length ranges. The cf‐nDNA and cf‐mtDNA with different fragment lengths differentially distributed in the CSF and serum of patients with brain disorders, which might serve as a biomarker of human brain diseases. We guessed that brain‐related diseases might lead to an increase of cfDNA release in CSF,^44^ and the released cfDNA might cross the blood‐brain barrier to blood, but the cfDNA might be obstructed by blood‐brain barrier when its fragment length is too long.^45^, ^46^ The cf‐nDNA could be obstructed by the blood‐brain barrier starting from medium fragment length, but cf‐mtDNA might be from long length. These results indicated that cf‐mtDNA could be easier to cross the blood‐brain barrier than cf‐nDNA in the same fragment length. The reason might be that the cf‐nDNA usually exists in the form of protein‐DNA complex, whereas cf‐mtDNA does not have this form,^47^ which results in a higher molecular weight of cf‐nDNA than that of cf‐mtDNA at the same DNA length.

## 5 CONCLUSIONS

According to the results of Agilent 2100 Bioanalyzer, qPCR, and ddPCR, there were short, medium, and long cfDNA fragments existing in CSF and serum. The copy numbers of long cf‐mtDNA, medium, and long cf‐nDNA in CSF were significantly higher than in paired serum by qPCR. The cf‐nDNA and cf‐mtDNA with different fragment lengths differentially distributed in the CSF and serum of patients with brain disorders, which might serve as a biomarker of human brain diseases.

## CONFLICT OF INTEREST

All authors in this study had no conflicts of interest.

## ETHICAL APPROVAL

The study was approved by the Ethics Committee of the hospital, and methodologies conformed to the standards set by the Declaration of Helsinki. All patients signed informed consent and volunteered to participate in the study.
