# Supplementary material for: Comparison of paired cerebrospinal fluid and serum cell‐free mitochondrial and nuclear DNA with copy number and fragment length
Source: J Clin Lab Anal. 2020 Feb 13;34(6):e23238. doi: 10.1002/jcla.23238 (PMC7307366; doi:10.1002/jcla.23238)
Supplement: Supplementary file 1 [file JCLA-34-e23238-s001.docx]

***Journal of Clinical Laboratory Analysis***

**Supplementary Material**

**Comparison of paired cerebrospinal fluid and serum cell-free mitochondrial and nuclear DNA with copy number and fragment length**

**Aolong Chen^1†^****∣Jun Li^2†^∣Lei Wang^1^∣Qin Huang^1^∣Jiajin Zhu^1^∣Shumeng Wen^1^∣Jianxin Lyu^1, 3^∣Wenhe Wu^1^**

^1^ Key Laboratory of Laboratory Medicine, Ministry of Education, Zhejiang Provincial Key Laboratory of Medical Genetics, School of Laboratory Medicine and Life Sciences, Wenzhou Medical University, Wenzhou, Zhejiang 325035, China

^2^ Department of Clinical Laboratory, Wenzhou People’s Hospital, Wenzhou, Zhejiang 325000, China

^3^ Hangzhou Medical College, Hangzhou, Zhejiang 310006, China

Correspondence

Jianxin Lyu, Key Laboratory of Laboratory Medicine, Ministry of Education, Zhejiang Provincial Key Laboratory of Medical Genetics, School of Laboratory Medicine and Life Sciences, Wenzhou Medical University, Wenzhou, Zhejiang 325035, China.

E-mail: jxlu313@163.com

Wenhe Wu, Key Laboratory of Laboratory Medicine, Ministry of Education, Zhejiang Provincial Key Laboratory of Medical Genetics, School of Laboratory Medicine and Life Sciences, Wenzhou Medical University, Wenzhou, Zhejiang 325035, China.

E-mail: wuwenhe2000@163.com or wwh2000@wmu.edu.cn

**^†^**Both authors contributed equally to this work


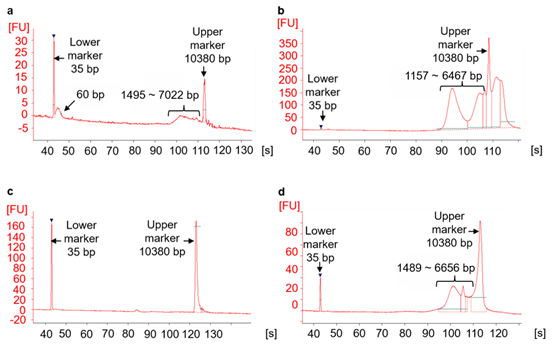


**FIGURE S1** Characterization of cfDNA fragment length by using Agilent 2100 Bioanalyzer. **a ~ d**, DNA fragment length in raw CSF solution without dilution **(a)**, raw serum solution without dilution **(b)**, raw CSF with 10-fold dilution **(c)** and raw serum with 10-fold dilution **(d)** from patient 3. X-axis represents the migration time of DNA fragments. Y-axis indicates fluorescence intensity. The lower marker is 35 bp and the upper marker is 10380 bp. The numbers above peaks indicate the length of DNA fragments.


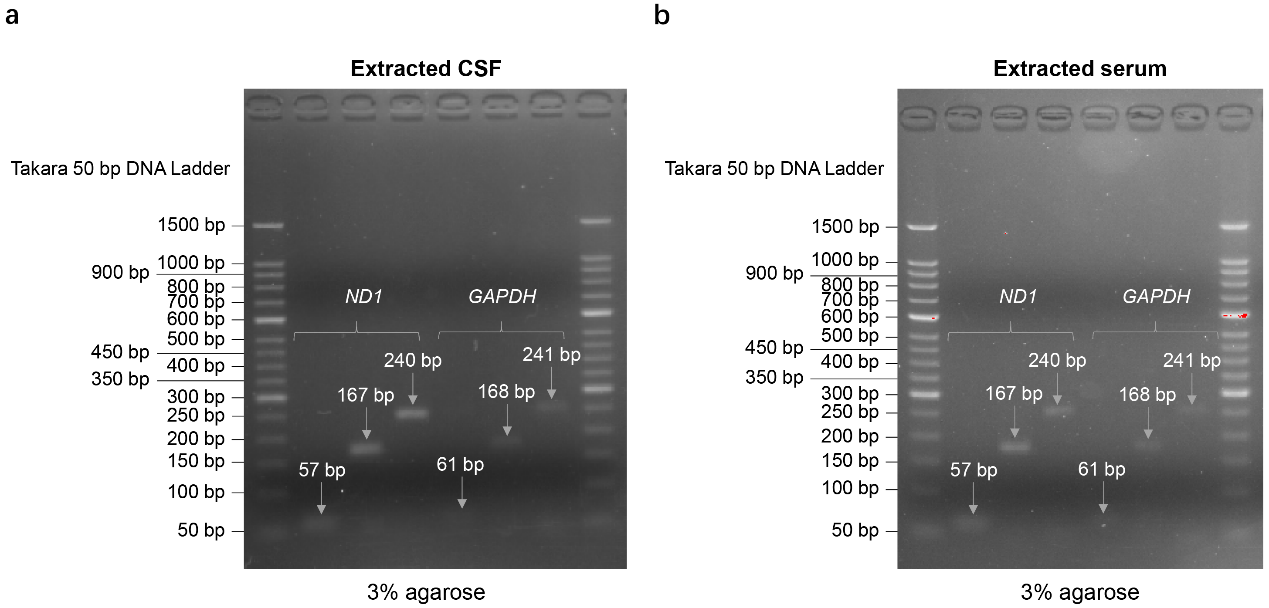


**FIGURE** **S2** Electrophoretic verification of qPCR products. **a**, The sample was extracted CSF added in qPCR system. b, The sample was extracted serum added in qPCR system. The marker was Takara 50 bp DNA Ladder ranging from 50 bp to 1500 bp. The DNA bands of sample wells were formed by the products after qPCR amplification with different primer pairs. The concentration of agarose we used was 3%.


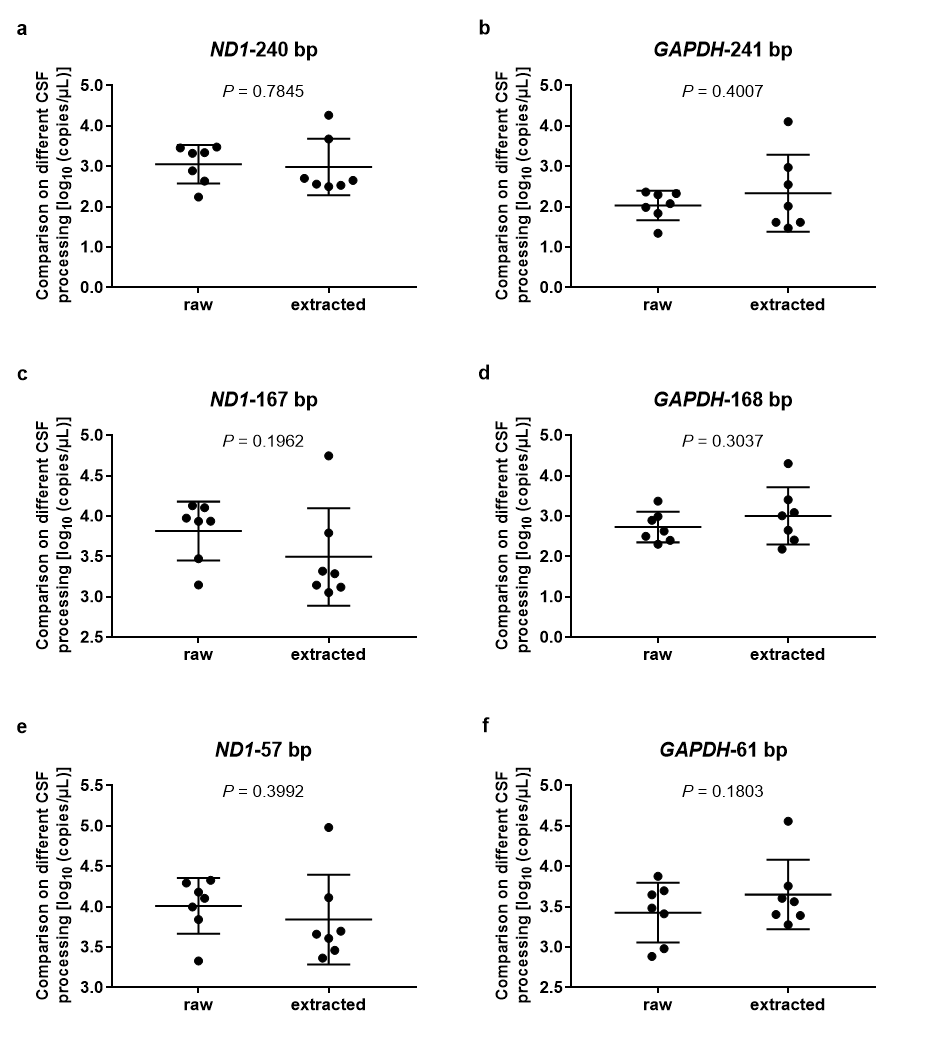


**FIGURE** **S3** Comparison between raw and extracted CSF by qPCR. The primer pairs were *ND1*-240 bp **(a)**, *GAPDH*-241 bp **(b)**, *ND1*-167 bp **(c),** *GAPDH*-168 bp **(d)**, *ND1*-57 bp **(e)** and *GAPDH*-61 bp **(f**), respectively. Data are presented as mean ± SD.


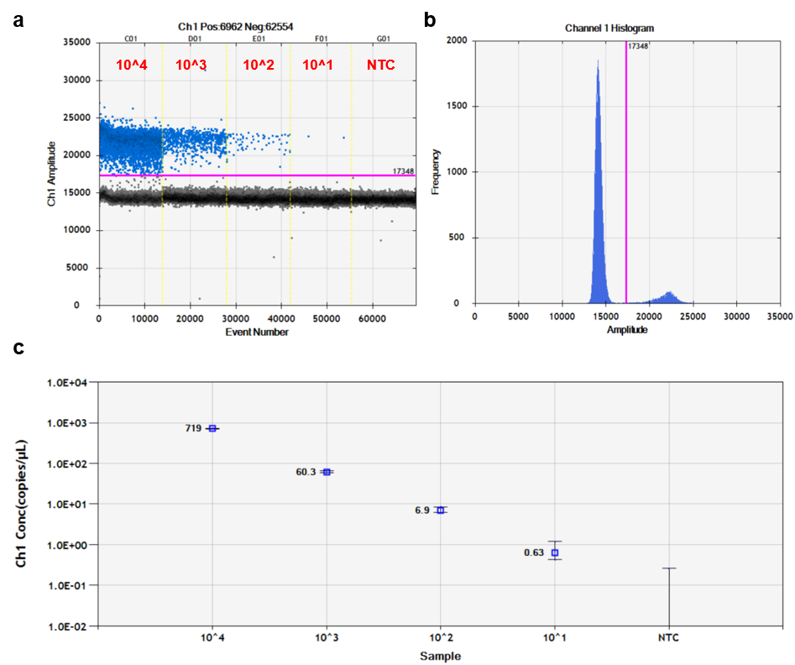


**FIGURE S4** Recombinant *GAPDH* plasmid detected by ddPCR. **a**, One-dimensional amplitude of droplets. X-axis represents reading number of droplets and Y-axis represents fluorescence signal intensity. Blue points: positive droplets; black points: negative droplets. Pink line: the threshold discriminates the positive and negative droplets. Yellow line: divisions of each samples. **b**, The histogram of plasmids. X-axis represents amplitude and Y-axis represents frequency distribution of droplets. Pink line: the threshold separates positive droplets from negative droplets. **c**, Linear correlation of serially diluted standards. X-axis represents serially standard plasmids of *GAPDH* gene. Y-axis represents log_10_ of copies/µL in reaction system.


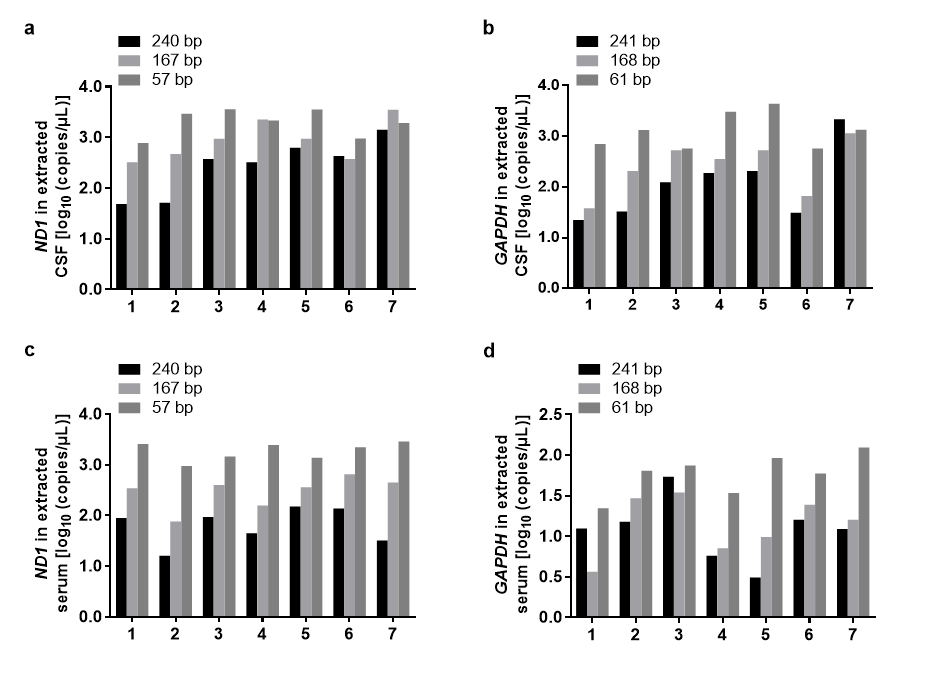


**FIGURE** **S5** Quantification of cfDNA copy number by ddPCR. **a**, **b**, Extracted CSF was amplified with primer pairs of *ND1* **(a)** and *GAPDH* **(b)**. **c**, **d**, Extracted serum was amplified with primer pairs of *ND1* **(c)** and *GAPDH* **(d)**. X-axis represents patients and Y-axis represents log_10_ of copies/µL in initial raw samples. Data are presented as mean ± SD.
